# Supplementary material for: Extreme heat prediction through deep learning and explainable AI
Source: PLoS One. 2025 Mar 20;20(3):e0316367. doi: 10.1371/journal.pone.0316367 (PMC11925282; doi:10.1371/journal.pone.0316367)
Supplement: Attachment — Submitted filename: Response to the Reviewers.pdf [file pone.0316367.s001.pdf]

Response to the Reviewers on Manuscript

Extreme Heat Prediction through Deep Learning and Explainable AI

Fatima Shafiq, Amna Zafar, Muhammad Usman Ghani Khan, Sajid Iqbal, Abdulmohsen Saud Albesher, Muhammad Nabeel Asghar

Dear Editors and Reviewers,

We extend our sincere gratitude for your valuable comments, which have greatly contributed to enhancing the quality of our manuscript. We have carefully addressed each reviewer's comment in our revisions and have made every effort to incorporate the suggestions provided by both the reviewers and editors to effectively demonstrate the strengths of our proposed work. Below, we provide a detailed summary of our responses and the corresponding changes made in the revised manuscript.

Kind regards,

The Authors

Reviewer 1

1. The results and discussion section does not have adequate depth and needs to be comprehensive and elaborated.

Response:

To address this, we have significantly enhanced the relevant sections of the manuscript. The updated content now includes comprehensive results for all our deep learning models, the regional threshold, and both the original and predicted extreme heat temperatures. Additionally, we have provided detailed interpretability analyses using XAI techniques, alongside a comparison with previous studies, to emphasize the effectiveness and novelty of our framework.

Please refer to Section 5 of the revised manuscript for these enhancements.

2. Provide brief background on explainable AI methods and its usefulness for complex models.

Response:

To address this, we have incorporated a brief background on explainable AI (XAI) methods to highlight their importance in dealing with the complexity of deep learning models. We have also included the suggested papers to illustrate the challenges posed by these "black box" models and how XAI techniques improve transparency and interpretability in the results.

Please refer to Section 3 in the revised manuscript for these additions.

3. Conclusion needs to be rephrased by providing main findings of the study

Response:

We have rephrased the conclusion to more clearly present the main findings of our study, including detailed results that highlight our key outcomes.

Please refer to Section 6 of the revised manuscript for these updates.

4. What is the reasoning behind using LIME and SHAP both? In many of the works, LIME has been compared with SHAP and sometimes there are inconsistencies between LIME and SHAP

Response:

We chose to use both LIME and SHAP in this study to take advantage of their complementary strengths. LIME is a model-agnostic local explainer, allowing us to analyze the impact of input variables on individual LSTM predictions for each observation period. On the other hand, SHAP provides global insights by assigning distinct values to each feature, offering a comprehensive understanding of feature importance across the entire model. Using both

methods allows us to capture both local and global perspectives, which is particularly important for analyzing weather-related time series data.

Please refer to Section 3 of the revised manuscript for these updates.

5. Results section should be significantly improved to have a scientifically thorough discussion.

Response:

To address this, we have significantly improved the Results section by providing a more scientifically thorough discussion. This updated section includes detailed results for our deep learning models, along with explanations of the results. We have also included the exact temperature predictions, as well as insights into model interpretability, to offer a comprehensive view of our findings.

To further enhance the value of our deep learning results, we have added an Experimentation section, which details the hyperparameters of each model and provides an overview of the training process for all models.

Please refer to Section 4 and Section 5 of the revised manuscript for these updates

6. Discussion should critically evaluate the predictions and explanations and compare findings and shortcomings with the related studies

Response:

For this, we have reorganized our manuscript to address this concern. We have combined the deep learning results, prediction outcomes, model explainability, and a comparison with previous studies into a single section titled "Results and Discussion". This section now provides a critical evaluation of the predictions and explanations, comparing our findings with related studies and discussing any shortcomings to offer a more comprehensive analysis.

Please refer to Section 5 of the revised manuscript for these updates

Reviewer 2

1. Though this is good work, I am afraid about novelty this can be addressed by referring recent past papers/ i suggest few recent past papers.

To address the concern regarding the novelty of our work, we have expanded the literature survey section to reference recent past papers, highlighting the gaps in current research. Additionally, we have added specific headings to emphasize the novelty of our study, including:

- **Limited Work on Regional Datasets**: There is minimal focus on extreme heat prediction datasets specific to South Asia. To address this, we collected the XWeather dataset from PMD, addressing a regional data gap in the field.

- **Limited Work on Explainable AI (XAI)**: XAI applications in extreme heat prediction are scarce. By using XAI, we improve model interpretability and identify critical factors influencing extreme heat events, enhancing transparency.
- **Limited Work on Deep Learning with XAI**: Few studies apply XAI to deep learning models for extreme heat prediction. To the best of our knowledge, no prior research has looked into the extraction of important features for extreme heat prediction using XAI, particularly in combination with DL models. Our study addresses this gap by using XAI to enhance the interpretability of DL predictions, making them more reliable.

These additions are intended to clarify the unique contributions of our work in comparison to existing studies.

Please refer to Section 2 and Section 5.3 of the revised manuscript for these updates.
